# Efficacy and Feasibility of the Epithelial Cell Adhesion Molecule (EpCAM) Immunomagnetic Cell Sorter for Studies of DNA Methylation in Colorectal Cancer

**DOI:** 10.3390/ijms15010044

**Published:** 2013-12-20

**Authors:** Alessandra Failli, Annalisa Legitimo, Francesca Migheli, Fabio Coppedè, John C. Mathers, Roberto Spisni, Paolo Miccoli, Lucia Migliore, Rita Consolini

**Affiliations:** 1 Department of Clinical and Experimental Medicine, University of Pisa, Pisa 56126, Italy; E-Mails: faillialessandra@gmail.com (A.F.); a.legitimo@ao-pisa.toscana.it (A.L.); 2 Department of Translational Research and New Technologies in Medicine and Surgery, Division of Medical Genetics, University of Pisa, Pisa 56126, Italy; E-Mails: francesca.migheli@for.unipi.it (F.M.); fabio.coppede@med.unipi.it (F.C.); lucia.migliore@med.unipi.it (L.M.); 3 Department of Laboratory Medicine, Pisa University Hospital (AOUP), Pisa 56126, Italy; 4 Human Nutrition Research Centre, Institute for Ageing & Health, Biomedical Research Building, Campus for Ageing & Vitality, Newcastle University, Newcastle upon Tyne NE4 5PL, UK; E-Mail: john.mathers@ncl.ac.uk; 5 Department of Surgery, Medical, Molecular, and Critical Area Pathology, University of Pisa, Pisa 56124, Italy; E-Mails: roberto.spisni@med.unipi.it (R.S.); paolo.miccoli@dc.unipi.it (P.M.)

**Keywords:** DNA methylation, epigenetics, epithelial cell adhesion molecule (EpCAM), immunomagnetic enrichment, colon cancer, pyrosequencing

## Abstract

The aim of this work was to assess the impact on measurements of methylation of a panel of four cancer gene promoters of purifying tumor cells from colorectal tissue samples using the epithelial cell adhesion molecule (EpCAM)-immunomagnetic cell enrichment approach. We observed that, on average, methylation levels were higher in enriched cell fractions than in the whole tissue, but the difference was significant only for one out of four studied genes. In addition, there were strong correlations between methylation values for individual samples of whole tissue and the corresponding enriched cell fractions. Therefore, assays on whole tissue are likely to provide reliable estimates of tumor-specific methylation of cancer genes. However, tumor cell tissue separation using immunomagnetic beads could, in some cases, give a more accurate value of gene promoter methylation than the analysis of the whole cancer tissue, although relatively expensive and time-consuming. The efficacy and feasibility of the immunomagnetic cell sorting for methylation studies are discussed.

## 1. Introduction

It is now widely accepted that cancer is a multi-step process resulting from the accumulation of both genetic and epigenetic alterations of the genome [1]. Gene mutations and epigenetic modifications were viewed initially as separate aetiological mechanisms for carcinogenesis but recent evidence points to crosstalk between these two mechanisms. Gene mutations may disrupt several epigenetic patterns and epigenetic modifications can drive genome instability and mutagenesis [2,3].

Colorectal cancer (CRC) is one of the commonest human cancers with over one million new cases diagnosed worldwide annually [4]. Most CRC cases (75%–80%) occur sporadically as a result of the accumulation of both mutations and epigenetic modifications of several genes [5], and large-scale DNA methylation studies suggest that CRC can be divided into at least three subtypes according to the patterns of DNA methylation and of mutations in key CRC genes [6,7].

DNA methylation involves covalent addition of methyl groups to the 5′ position on cytosine residues, usually in cytosine-phosphate-guanine (CpG) dinucleotides, and is one of the most studied epigenetic marks in CRC [8]. Methylation of CpG islands (domains unusually enriched with CpG dinucleotides) in the promoter region of a gene can induce chromatin conformational modifications and inhibit the access of the transcriptional machinery, thus altering gene expression levels. Promoter hypermethylation is commonly associated with gene silencing and promoter demethylation with gene expression. The ever-growing number of genes that show epigenetic alterations in cancer emphasizes the crucial role of these epigenetic alterations, and particularly of DNA methylation, for future diagnosis, prognosis and prediction of response to therapies [9]. Therefore, active research is currently ongoing to develop rapid, cost effective and reproducible tools for the detection of epigenetic marks [9]. Most of the currently available techniques are based on DNA analysis following sodium bisulfite treatment that converts unmethylated cytosines to uraciles, leaving methylated cytosines unchanged. At present, pyrosequencing of bisulfite treated DNA is considered as a gold standard technique for the quantification of DNA methylation [10]. There is now good evidence of differential DNA methylation patterns between normal cells in tissues and cancerous cells in equivalent tissues [11]. Human solid tumors are characterized by phenotypically heterogeneous populations of both normal and malignant cells, with varying degrees of differentiation and tumor initiating potential. Therefore, investigation of DNA methylation patterns in samples containing such mixed tissue might be confounded due to the presence of normal cells with different methylation patterns.

The epithelial cell adhesion molecule (EpCAM; CD326) is a transmembrane glycoprotein, highly overexpressed on most carcinomas, and its downregulation inhibits the oncogenic potential of multiple tumor types [12]. EpCAM is expressed at a high level and frequency in colon cancer tissues (in 97%) and in most human adenocarcinomas [13–15]. Recently, EpCAM has become of interest because it is a signal transducer [12,16], and a potential marker of cancer-initiating cells [17] whose role in the development of cancer and in tumor progression depends on the tumor type [12]. In certain tumor types, EpCAM overexpression is linked to advanced stage of disease and worse overall survival, suggesting that EpCAM may have utility as a potential prognostic marker [18].

In the present study, we aimed to compare the methylation profiles of DNA extracted from an EpCAM-enriched cell fraction and from non-enriched CRC cells (whole tumor DNA) to determine whether such cellular enrichment with tumor cells would increase the sensibility of the results. For this purpose, we quantified DNA methylation profiles of four CRC-related genes (*APC*, *MGMT*, h*MLH1*, *CDKN2A/p16*) in DNA obtained from human CRC tissues.

## 2. Results and Discussion

### 2.1. Results

#### 2.1.1. Patient Characteristics

The study included 24 patients with primary colorectal adenocarcinoma. Median age of CRC patients was 73 years (range 47–91) with 10 females and 14 males. Five of 24 patients presented with metastatic disease to the liver and were classified as TNM stage IV; 6 patients were TNM stage III, 9 and 4 patients were TNM stages II and I, respectively. The characteristics of the patients are summarized in Table 1.

#### 2.1.2. Recovery of EpCAM+ Cells

The cell recovery rate was calculated as the percentage of the EpCAM+ cell number obtained from immunomagnetic selection divided by the total number of cells in the suspension before immunomagnetic selection. The median recovery for EpCAM+ (tumor) cells was 36.9% (range 10.1%–94.3%; Table 2). There were no significant correlations between recovery rate of EpCAM positive cells with age or gender of the patients or with the clinical stage or with histologic features of the tumor (data not shown).

#### 2.1.3. DNA Methylation

**Figure 1.**
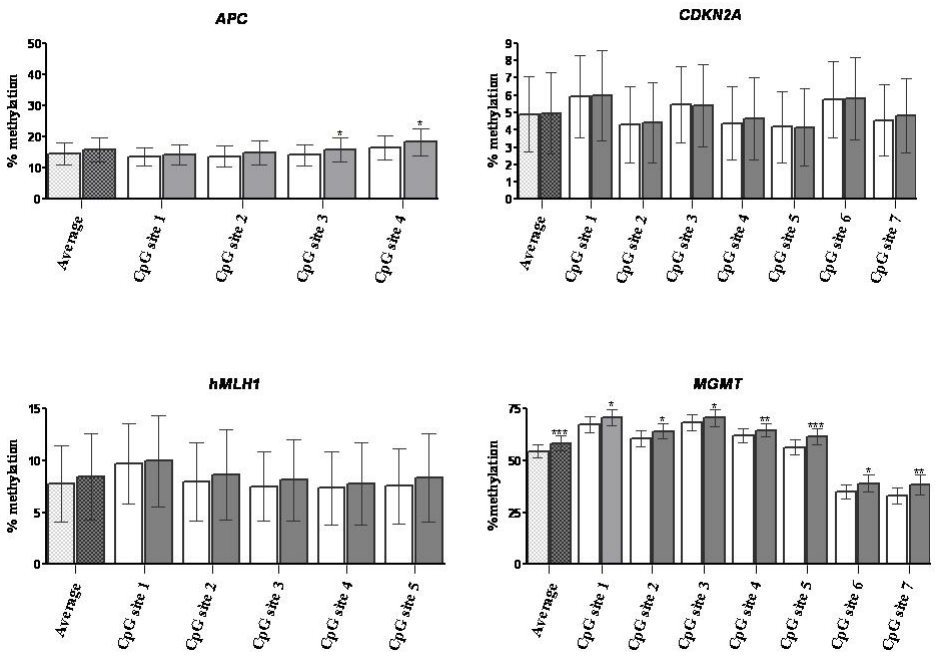
Comparison of the DNA methylation status between EpCAM non-enriched and enriched samples. Methylation of each gene (Average) and of the individual CpG sites was evaluated in 24 CRC patients. The bars represent the mean of the methylation values of each subject analyzed ± the S.E.M. Dark bars indicate EpCAM-enriched samples. Significant differences are indicated: * *p* < 0.05, ** *p* < 0.01, *** *p* < 0.001.

The methylation of specific CpG sites (4–7 sites per gene) within the promoter regions of all four genes (*APC*, *CDKN2A*, h*MLH1*, *MGMT*) was quantified by pyrosequencing. To avoid potential confounding, both cell samples (EpCAM enriched and non-enriched cell populations) for each patient were processed and analyzed simultaneously (Figure 1). As expected, there were considerable differences in CpG methylation among genes (highest for *MGMT* and lowest for *CDKN2A*). For *APC*, the % methylation increased slightly from CpG 1 to CpG 4. Averaged across all 4 CpG sites, there was no significant difference in % methylation between EpCAM enriched and non-enriched cell populations (15.9% ± 18.2% and 14.6% ± 16.4% respectively). However, there was significantly higher methylation at CpG sites 3 and 4 for the enriched samples than the non-enriched ones (15.9% ± 18.5% and 18.5% ± 20.7% *versus* 14.2% ± 16.7% and 16.7% ± 18.7%, respectively; *p* < 0.05).

Methylation of the *CDKN2A* promoter was relatively low (4%–6%) and at each of the seven CpG sites analyzed there were no significant differences between the EpCAM-enriched and non-enriched samples.

Similarly, for the five CpG sites interrogated within the h*MLH*1 promoter there were no significant differences in methylation for the EpCAM+ cells compared with the whole tumor cell suspension.

The *MGMT* promoter was heavily methylated at all CpG sites in all patients and we observed significantly higher methylation in EpCAM enriched samples compared with the corresponding non-enriched samples. This effect was evident at each of the seven CpG sites individually and for the mean across all seven sites (mean methylation was 58.5% ± 17.8%, range: 26.6%–86.6% *versus* 54.7% ± 16.0%, range: 24.2%–78.3%; *p* = 0.0002 for EpCAM+ and non-enriched cell fractions, respectively (Figure 1).

Using mean data for all CpG sites within each gene promoter, Table 2 compares the methylation percentages of both enriched and not enriched samples for each of the 4 gene promoters in relation to the clinical stage. There were significant differences in methylation content between the enriched and the non-enriched samples only for tumors in stages II and III for the *MGMT* promoter (58.4% ± 17.6% *versus* 54.2% ± 14.4%, *p* < 0.05 and 50.4% ± 18.3% *versus* 47.8% ± 19.1%, *p* < 0.01, respectively).

**Figure 2.**
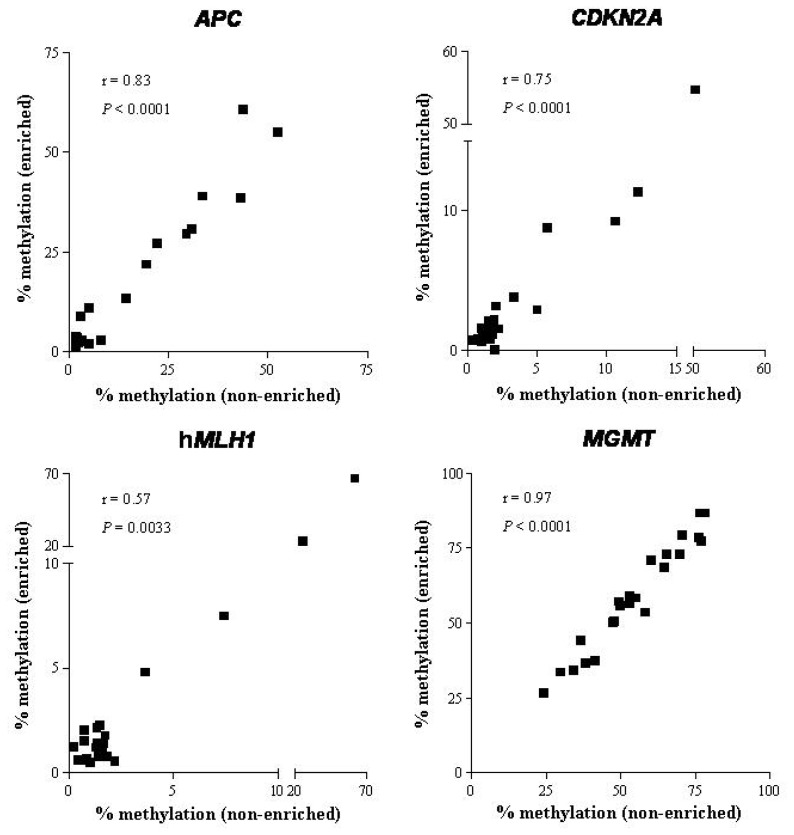
Correlation between the methylation values of the enriched and non-enriched samples. The panels represent the average percent methylation for all CpG loci analyzed in non-enriched (*X* axis) and in enriched samples (*Y* axis). Spearman’s correlation coefficients and *p*-values are shown.

Correlation coefficients between enriched and non-enriched cell fractions were high for methylation of *APC*, *MGMT* and *CDKN2A* genes (Spearman *r* = 0.83, 0.97 and 0.75, respectively; *p* < 0.0001) and moderate for h*MLH1* gene (Spearman *r* = 0.57, *p* = 0.0033) (Figure 2).

**Figure 3.**
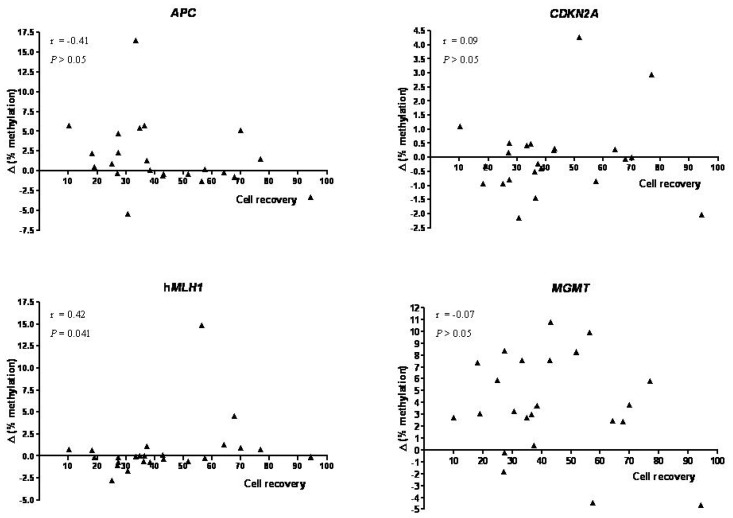
Correlation between cell recovery and values obtained by the difference in DNA methylation between the enriched and non-enriched cell fractions. Tumor cell recovery (*X* axis) was obtained by the ratio between the EpCAM positive fraction and the whole tumor sample. Δ methylation (*Y* axis) represents the values obtained by the difference in average percent methylation for all CpG loci analyzed between the EpCAM-enriched and non-enriched samples. Spearman’s correlation coefficients and *p*-values are shown.

Finally, we investigated the possibility that the methylation differences between the enriched and the non-enriched samples might be correlated with EpCAM+ cell recovery (Figure 3). We found no convincing relationships for any of the four genes. However, for h*MLH1*, there was a weak, but significant, correlation (Spearman *r* = 0.42; *p* = 0.04). This apparent relationship for the h*MLH1* gene only depended heavily on just one tumor specimen displaying high methylation content with little evidence of any relationship for any of the other samples. The outlier was a tumor characterized by high necrotic component (patient in stage II); indeed, after removal of the sample data assumed a normal distribution and there was no significant correlation (Pearson *r* = 0.18; *p* > 0.05).

### 2.2. Discussion

DNA methylation is one of the most widely studied epigenetic marks in CRC and there is increasing interest in the identification and characterization of epigenetic signatures for CRC diagnosis, staging, tendency to metastasis, prognosis, and response to treatment [8,19]. However, resected CRC specimens are heterogeneous and include areas of non-malignant mucosal and connective tissue [20]. Given the differences in methylation between cell and tissue types [21], it is possible that the inclusion of stromal and other cells within a whole resected tumor specimen might confound analysis of the methylation content of the CRC tumor cells. This could be a particular problem for tumor suppressor genes which are usually unmethylated in normal tissue but can be heavily methylated in tumor cells. Since the percentages of stroma *versus* tumor epithelium vary widely among patients, this high degree of heterogeneity could add further complexity to comparisons of different tumor samples [22].

Immunomagnetic cell sorting using a tumor-specific antibody might overcome these problems by providing a more homogeneous sample of cells [23,24].

The epithelial cell adhesion molecule (EpCAM) is a transmembrane glycoprotein only expressed in epithelium and neoplasias derived from epithelia. It was first described as a dominant antigen in human colon carcinoma due to its strong expression in colon adenocarcinoma (close to 100%).

In this work, we tested the hypothesis that EpCAM tumor cell enrichment of the CRC resected specimens would provide better estimates of the methylation of tumor-related genes in the sample compared with analysis of whole specimen. By comparing the methylation profile of the EpCAM+ enriched fraction with the non-enriched fraction from the same resected human CRC specimens, we showed that significant differences were obtained for all the CpG sites within the promoter region of *MGMT*—a gene showing high methylation values in our cohort. In addition, we observed significant differences also for 2 out of 4 CpG islands in the promoter of the *APC* gene (CpG site 3 and 4). When averaged across all CpG sites investigated, only the *MGMT* promoter region showed significant differences in methylation between the EpCAM-enriched and non-enriched cell fractions. By contrast, there were no average methylation differences between the two cell fractions for the other three genes that we investigated. Moreover, we did not detect convincing relationships between the EpCAM+ cell recovery and the difference in DNA methylation between the enriched and non-enriched cell fractions.

Present results, albeit obtained by means of the analysis of only four genes in a relatively small group of CRC patients, suggest that also low amounts of gene promoter methylation can be detected either with or without EpCAM tumor cell enrichment; however, the analysis of the EpCAM enriched cell fraction provides a more accurate value of gene methylation than the analysis of the whole DNA sample, particularly in the case of high levels of promoter methylation such as those observed for *MGMT* or *APC* CpG site 3 and 4. Therefore, as DNA methylation percentage from EpCAM enriched samples is more specific for tumor methylation than that obtained from non-enriched samples, we believe that this procedure represents a valuable approach for the investigation of the “tumoral target”, and particularly in those circumnstances where the tumoral component is supposed to be relatively lower than the non-tumoral one, or if a precise value of methylation is required for diagnostic, research or therapeutic purposes.

EpCAM may thus be an ideal tumor antigen candidate to detect circulating and metastasizing cancer cells. Although methylation of cancer-related genes is recognized as a promising biomarker for early detection and prognosis estimation [25,26], it has been scarcely investigated in Circulating Tumor Cells (CTCs). Recently, Chimonidou *et al.* demonstrated that DNA methylation of tumor suppressor and metastasis suppressor genes exists in CTCs [25].

As EpCAM is also abundantly expressed in cancer stem cells [18,27,28] it might be an ideal tumor antigen candidate to detect circulating and metastasizing cancer cells by immunomagnetic sorting [18,29,30], useful to elucidate the interplay between epigenetic gene silencing and other tumorigenic processes in CTCs for the understanding of tumor evolution and metastasis.

Additionally, reliable detection of micrometastatic cells in the sentinel lymph node is a subject of great clinical interest, and several different protocols aimed to identify epithelial cells within the lymphatic basin are currently in use in breast cancer. As these cells may exist in low concentrations, their identification and isolation represents a difficult task [23].

Therefore, we think that the effort to apply this expensive and laborious procedure can be employed in selected experimental settings, particularly in the investigation of the CTCs: The study of methylation profile of the enriched CTCs may add a new dimension to the malignant nature and metastatic potential of these cells.

## 3. Experimental Section

### 3.1. Tissue Samples

Colorectal tissue samples were collected, with informed consent, from 26 Italian patients undergoing curative bowel resection at the Cisanello Hospital (Department of Surgery, Medical, Molecular, and Critical Area Pathology, University of Pisa, Pisa, Italy). Individuals who met the study eligibility criteria and provided written informed consent were enrolled. None of the patients received preoperative chemotherapy or radiotherapy.

The study was performed according to the principles of the Declaration of Helsinki and was approved by the Clinical Research Ethics Committee of the Pisa University Hospital.

All samples were evaluated and diagnosed histologically by an expert pathologist, and representative tissue sections were selected for DNA extraction and further molecular analyses.

### 3.2. Generation of a Single Cell Suspension from Colorectal Cancer Tissues

Cells were derived from biopsies as previously described [31]. After resection, intestinal tissue was cut lengthwise, deeply into the submucosal layer, avoiding fistulas and necrosis. After extensive washing with saline solution, the fragments were dissected into wedge-shaped pieces and immediately placed in a sterile container containing a basic transport medium (RPMI; Gibco Laboratories, Grand Island, NE, USA). Tumor samples were washed in RPMI medium supplemented with concentrated antibiotics (penicillin (500 U/mL), streptomycin (500 μg/mL), gentamicin (100 μg/mL), amphotericin B (12.5 μg/mL) and metronidazole (5 μg/mL)) and then minced with a medical scalpel into 0.5–2.0 mm^3^ fragments excluding macroscopic excess fat and necrotic tissues. Fragments were immediately enzymatically digested with 5–10 mL of bovine collagenase type H (Sigma-Aldrich, Dublin, Ireland) at a concentration of 1.5 mg/mL and shaken by an agitator for about 6 h at 37 °C. Successful enzymatic digestion was assessed by examining an aliquot of the cell suspension under an inverted microscope. The suspension was then filtered through a double layer of sterile gauze and washed with medium containing foetal bovine serum to inactivate collagenase.

Cell viability was assessed by the trypan blue dye exclusion method and the number of cells was determined by hemocytometer counting.

Each cell suspension was divided into two aliquots: one was immediately stored at −20 °C until processing and the other one was used for the tumor cell enrichment procedure.

### 3.3. Tumor Cell Enrichment with Anti-EpCAM Antibodies

Tumor cells were isolated from the cell suspensions obtained as described above using immunomagnetic beads coated with anti-EpCAM (CD326) antibodies using the manufacturer’s protocol (Miltenyi Biotec GmbH, Bergisch Gladbach, Germany).

Briefly, the cell suspension was diluted with buffer (0.5% foetal calf serum, 2 mM ethylenediamine tetraacetic acid (EDTA) in phosphate buffered saline (PBS), pH 7.2) and incubated with the anti-EpCAM antibody-loaded microbeads for 30 min at 2–8 °C, then washed with buffer solution. Then the cell suspension was transferred to a column placed in the magnetic field of a MACS Separator (Miltenyi Biotec, Bergisch Gladbach, Germany). This allowed separation of the magnetically labeled CD326+ cells which were retained on the column whilst the unlabeled cells (CD326−) ran through the column. The column was then removed from the magnetic field and the CD326+ cells were eluted and collected. The recovered CD326+ cells were enumerated and maintained at −20 °C. Tumor cell recovery was calculated as percentage of the CD326+ (EpCAM) cell number obtained from immunomagnetic selection divided by total number of cells in the tumor cell suspension before immunomagnetic selection.

### 3.4. Extraction of Genomic DNA

Genomic DNA was extracted using the QIAmp DNA blood Mini Kit (Qiagen, Milan, Italy) according to the manufacturer’s instruction. The extracted DNA was quantified using a Nano Drop ND 2000c spectrophotometer (NanoDrop Thermo scientific, Wilmington, DE, USA).

### 3.5. Bisulfite Modification

Sodium bisulfite modification was performed using the EZ DNA MethylationGold Kite (Zymo Research, Orange, CA, USA), according to the manufacturer’s instructions. Bisulfite-treated DNA was eluted in 10 μL of elution buffer.

### 3.6. Pyrosequencing

DNA methylation was measured performing the amplification of bisulfite modified DNA, followed by pyrosequencing. Genomatix software (http://www.genomatix.de) Gene2Promoter was employed to identify the promoter regions of interest and PSQ Assay Design software (Biotage, Uppsala, Sweden) was used to design the corresponding primer sets. Bisulfite modified (BM) 0% and 100% methylated DNA were diluted to produce DNA mixtures with defined methylation content (0%, 25%, 50%, 75%, 100%) which were used subsequently for PCR and pyrosequencing validation. The standard conditions for the PyroPCR were: 12.5 μL Taq Mastermix (Qiagen, Milan, Italy), 10 pmol of each primer and 50 ng of BM DNA in a total volume of 25 μL. The PyroPCR temperature profile was the following: 95 °C for 15 min, 94 °C for 15 s, Ta for 30 s, 72 °C for 30 s (Repeat steps 2, 3, 4 × 50 times) and 72 °C for 10 min. Table 3 shows the pyrosequencing conditions (primers, annealing temperature and CpG sites analyzed) for the studied genes (*CDKN2A*, *APC*, *MGMT* and h*MLH1*). Pyrosequencing was performed at the Human Nutrition Research Centre, Institute for Ageing & Health, Newcastle University, Newcastle upon Tyne, UK. A fully methylated and non-methylated control DNA was included in each run to verify the validity of pyrosequencing results. Methylation sensitive-high resolution melting technique (MS-HRM) was performed in a subgroup of 10 subjects to validate pyrosequencing data (Details on the comparison of pyrosequencing and MS-HRM for *APC* and *CDKN2A* genes can be found in reference [19]).

### 3.7. Statistics

All numerical data were expressed as mean, and the standard deviation (SD) was calculated. The data from EpCAM-enriched and non-enriched cell fractions were compared using Student’s *t* test (for continuous, normally distributed data) or Wilcoxon signed-rank test (for non-normally distributed paired data). Two-tailed *p*-values were considered statistically significant at *p* < 0.05. Statistical testing was carried out using GraphPad Prism 4.0 (GraphPad Software Inc., San Diego, CA, USA).

## 4. Conclusions

Tumor cell tissue separation using immunomagnetic beads is relatively expensive and time-consuming. However, our findings suggest that, in some cases, it could give a more accurate value of gene promoter methylation than the analysis of the whole cancer tissue, and might therefore be of value in those circumstances where the tumoral component is supposed to be lower than the non-tumoral one.

## Figures and Tables

**Table 1.** Patient and tumor characteristics.

Characteristic	No. of patients
Age (range years)	

≤50 (47–50)	3
51–69 (53–65)	5
70–79 (70–76)	5
≥80 (81–91)	11

Gender	

Female	10
Male	14

Stage	

I	4
II	9
III	6
IV	5

Primary tumor site	

Colon dx	15
Colon sx	9

Tumor differentiation	

Well	0
Moderate	15
Poor	9

Additional pathologic characteristics	

Signet ring cell	0
Lymphatic invasion	11
Vascular invasion	8
Tumor budding	12

**Table 2.** DNA methylation in whole tumor cell suspension (non-enriched) and in epithelial cell adhesion molecule (EpCAM)+ (tumor) cells (enriched). Cell recovery: Percentage of the EpCAM (CD326)+ cell number obtained from immunomagnetic selection divided by total number of cells in the tumor cell suspension before immunomagnetic selection. Data are expressed as Median (in brackets range).

Clinical stage	Recovery	*APC*		*CDKN2A*		h*MLH1*		*MGMT*	

		Non-enriched	Enriched	Non-enriched	Enriched	Non-enriched	Enriched	Non-enriched	Enriched
I (*n* = 4)	64.4% (27.3%–95.3%)	13.7% (2.1%–31.0%)	15.3% (1.8%–30.7%)	4.1% (2.0%–50.4%)	5.1% (0%–54.7%)	1.2% (0.7%–1.4%)	0.7% (0.5%–2.1%)	64.4% (49.9%–78.3%)	67.4% (53.4%–83.6%)
II (*n* = 9)	36.5% (10.1%–70.0%)	5.2% (2.1%–33.8%)	11% (2.4%–38.9%)	1.8% (0.8%–12.3%)	1.8% (0.7%–11.3%)	1.7% (0.3%–64.7%)	1.5% (0.6%–79.6%)	53.1% (36.6%–76.7%)	59.0% * (36.4%–86.6%)
III (*n* = 6)	36.1% (18.9%–64.2%)	2.5% (2.0%–14.5%)	2.7% (2.3%–19.9%)	1.1% (1.0%–1.9%)	1.4% (0.7%–2.1%)	1.6% (0.7%–3.7%)	1.8% (0.6%–4.8%)	50.4% (24.2%–76.9%)	53.5% ** (26.6%–77.3%)
IV (*n* = 4)	38.1% (27.3%–67.7%)	23.8% (1.8%–55.6%)	28.8% (1.2%–60.6%)	1.8% (0.5%–3.4%)	1.5% (0.7%–3.8%)	1.5% (0.5%–61.8%)	1.2% (0.6%–66.4%)	57.4% (34.4%–76.2%)	64.9% (34.2%–78.6%)

* *p* < 0.05

** *p* < 0.01 differences calculated between the enriched and non-enriched samples.

**Table 3.** Pyrosequencing conditions and analyzed sequences.

Genes	Primer pyrosequencing	*T*a	RefSeqGene	CpG sites analyzed
*APC*	F-TATTAATTTTTTTGTTTGTTGGGGA	55 °C	NG_008481.4	C/TGGAGTGC/TGGGTC/TGGGAAGC/TGG
	R-AACTACACCAATACAACCACATATC			AGAGAGAAGTAGTTGTGTAATTC/TGTTG
	Sequencing primer: GGGGTTTTGTGTTTTATTG			GATGC/TGGATTAGGGC/TGT

*CDKN2A*	F-AGAGGATTTGAGGGATAG	50 °C	NG_007485.1	GAGGGTGGGGC/TGGATC/TGC/TGTGC/TG
	R-AATTCCCCTACAAACTTC			TTC/TGGC/TGGTTGC/TGGAGAGGGGGAGAGT
	Sequencing primer: GGGTTGGTTGGTTATTA			AGGTAGC/TGGGC/TGGC/TG

h*MLH1*	F-GGTTATAAGAGTAGGGTTAA	45 °C	NG_007109.2	TTC/TGTATTTTTC/TGAGTTTTTAAAAAC/TGA
	R-ATACCAATCAAATTTCTC			ATTAATAGGAAGAGC/TGGATAGC/TGATTTTT
	Sequencing primer: TGTTTTTATTGGTTGGATAT			AAC/TGC/TGTAAGC/TGTA

*MGMT*	F-AGTTTTTTTGGTGGATATA	47 °C	NP_002403.2	TC/TGC/TGTTTC/TGGGTTTAGC/TGTAGTC/TGT
	R-TACCTTTTCCTATCACAA			TTC/TGAGTAGGATC/TGGGATTTTTATTAAG
	Sequencing primer: TTTAGGAGGGGAGAGAT			
